# The Use of the Shock Index to Classify Patients During Mass-Casualty Incident Triage

**DOI:** 10.1017/S1049023X25101209

**Published:** 2025-06

**Authors:** David Jerome, David W. Savage, Matthew Pietrosanu

**Affiliations:** 1. Clinical Assistant Professor, Department of Family Practice, University of British Columbia, Vancouver, BC, Canada; Assistant Professor, Division of Clinical Sciences, NOSM University, Thunder Bay, ON, Canada; Assistant Adjunct Professor, Department of Family Medicine, University of Alberta, Edmonton, AB, Canada; ORCID: 0002-1831-4273; 2. Assistant Professor, Division of Clinical Sciences, NOSM University, Thunder Bay, ON, Canada; ORCID: 0000-0003-2837-3127; 3. Department of Mathematical and Statistical Sciences, University of Alberta, Edmonton, AB, Canada; ORCID: 0003-2349-0535

**Keywords:** Disaster Medicine, mass casualty, prehospital, triage

## Abstract

**Objectives:**

During mass-casualty incidents (MCIs), prehospital triage is performed to identify which patients most urgently need medical care. Formal MCI triage tools exist, but their performance is variable. The Shock Index (SI; heart rate [HR] divided by systolic blood pressure [SBP]) has previously been shown to be an efficient screening tool for identifying critically ill patients in a variety of in-hospital contexts. The primary objective of this study was to assess the ability of the SI to identify trauma patients requiring urgent life-saving interventions in the prehospital setting.

**Methods:**

Clinical data captured in the Alberta Trauma Registry (ATR) were used to determine the SI and the “true” triage category of each patient using previously published reference standard definitions. The ATR is a provincial trauma registry that captures clinical records of eligible patients in Alberta, Canada. The primary outcome was the sensitivity of SI to identify patients classified as “Priority 1 (Immediate),” meaning they received urgent life-saving interventions as defined by published consensus-based criteria. Specificity, positive predictive value (PPV) and negative predictive value (NPV) were calculated as secondary outcomes. These outcomes were compared to the performance of existing formal MCI triage tools referencing performance characteristics reported in a previously published study.

**Results:**

Of the 9,448 records that were extracted from the ATR, a total of 8,650 were included in the analysis. The SI threshold maximizing Youden’s index was 0.72. At this threshold, SI had a sensitivity of 0.53 for identifying “Priority 1” patients. At a threshold of 1.00, SI had a sensitivity of 0.19.

**Conclusions:**

The SI has a relatively low sensitivity and did not out-perform existing MCI triage tools at identifying trauma patients who met the definition of “Priority 1” patients.

## Introduction

Prehospital triage is performed during mass-casualty incidents (MCIs) to ensure that patients with life-threatening injuries receive urgent medical attention while limiting the application of clinical resources to less critical cases. Triage tools provide a systematic approach to rapidly assess and prioritize patients in these complex environments. Many formal triage tools have been developed, including Simple Triage and Rapid Assessment (START);^1^ JumpSTART (pediatric version of START);^2^ Sort, Assess, Life-saving Interventions, Treatment/Transport (SALT);^3^ Rapid Assessment of Mentation and Pulse (RAMP);^4^ Modified Physiological Triage Tool (MPTT);^5^ Battlefield Casualty Drills (BCD);^6^ and Major Incident Triage Tool (MITT).^7^ Recent work has demonstrated that these tools have an unacceptably low sensitivity in identifying patients who require urgent life-saving interventions.^8–10^ Due to the limitations of these tools, there is an on-going need to identify alternative methods for triaging patients during MCIs.

The calculation of a patient’s Shock Index (SI), defined as the ratio of heart rate (HR) to systolic blood pressure (SBP), offers a quick and efficient means of patient assessment. Previous work has shown that an elevated SI identifies patients at increased risk of hospital admission,^11^ in-patient mortality,^11,12^ blood transfusion,^12,13^ and hemodynamic instability.^14^ An elevated SI has also been shown to be an indicator of injury severity in trauma patients.^15^ Calculation of SI may therefore be a method for efficient patient triage during MCI events.

This study utilized clinical data from a provincial trauma registry in Alberta, Canada to measure the performance of SI in identifying trauma patients who received urgent life-saving interventions and compared the performance of SI to existing formal triage tools. It was hypothesized that an elevated SI would have a high sensitivity in identifying trauma patients who required urgent life-saving interventions, meeting a reference standard definition of a “Priority 1” patient, and that the SI would out-perform formal triage tools currently in use.

## Methods

This retrospective health records review retrieved clinical data from the Alberta Trauma Registry (ATR; Alberta Health Services; Edmonton, Alberta, Canada). The ATR is a web-based provincial trauma registry from Alberta, Canada that stores prehospital and in-hospital clinical data of eligible trauma patients.^16^ The registry enrolls all patients who are admitted to one of the province’s ten trauma centers with an Injury Severity Score (ISS) of 12 or more, as well as those who are pronounced dead in the emergency department (ED) of a trauma center. All patients admitted to a trauma center with penetrating trauma are enrolled in the registry as well.^16^ Staff at each trauma center update the registry on a daily basis. Records were retrieved for all patients enrolled in the registry from January 1, 2015 through December 31, 2019. Clinical data from the ATR were extracted by the database managers and provided to the study team in a password-protected Excel (Microsoft Corp.; Redmond, Washington USA) file.^17^ Patient-identifying data were removed and replaced with anonymous study IDs by the primary author before the file was shared with the rest of the research team.

Vital signs reported in the ATR were used to calculate each patient’s SI. The earliest clinical information documented in the ATR for each patient was utilized (eg, either in the prehospital setting, in the ED of a referring hospital, or in the ED of the trauma center).

The definition of a “Priority 1 (Immediate)” patient from a previously published, consensus-based definition of triage categorizations was used as the reference standard for critically injured patients who should be prioritized during MCI events.^18^ This definition was established by expert consensus in order to provide a reference for future evaluation of MCI triage systems and has subsequently been used by many studies assessing MCI triage tool performance.^8–10,19^ The inclusion criteria to be classified as a “Priority 1” patient includes having uncontrolled hemorrhage on presentation to an ED or requiring specific critical care interventions within two-to-four hours from presentation to the ED.

Due to how clinical data were recorded in the ATR, some assumptions had to be made in order to match the available clinical data with the criteria of the reference standard. For example, it was assumed that if the ATR documented that a patient had a chest tube placed either before arriving at the trauma center, or within the ED of the trauma center, then they met the reference standard criteria for “chest tube placed within two hours of arrival at hospital.” The assumptions employed are described in detail in the Supplementary Materials, available online only.

All records extracted from the ATR were included in the analysis unless missing clinical data prevented calculation of the patient’s SI or determination of their reference standard triage category. Excel functions were applied to calculate each patient’s SI and to identify which patients met the reference standard definition of a “Priority 1” patient. A receiver operating characteristic (ROC) curve analysis was performed to evaluate the discriminative value of SI. An optimal threshold for SI was determined using Youden’s index (ie, by maximizing the sum of sensitivity and specificity). The performance of SI was measured both at this optimal threshold and at a threshold of 1.00. A threshold of 1.00 was included as it can be easily and quickly recognized by observing that a patient’s HR is greater than their SBP. This threshold has been previously identified as an indicator that trauma patients require a massive blood transfusion.^13^

The pre-determined primary outcome was the sensitivity of SI (at both thresholds) for identifying patients who met the reference standard criteria of being a “Priority 1” patient. Specificity, positive predictive value (PPV), and negative predictive value (NPV) were calculated as secondary outcomes. Corresponding confidence intervals (CI) were calculated using Wilson’s score method with a continuity correction. Statistical analysis was completed using R (version 3.6.3; R Foundation for Statistical Computing; Vienna, Austria)^20^ and the pROC package (version 1.17.0.1).^21^

In order to compare the performance of SI to existing MCI triage tools, data from a previously published study that measured the performance of formal triage tools using the same reference standard were employed.^8^ That study assessed the performance of START, Jump START, SALT, RAMP, BCD, MITT, and MPTT. The previous investigation reported the same primary and secondary outcomes, analyzed data extracted from the ATR for the same date range, and used the same reference standard as this study. The use of the same data source, outcomes, and reference standard between the two studies limits the introduction of bias and allows for the comparison of performance metrics (eg, sensitivity) between the studies.

This study’s design followed the Recommendations for Reporting the Results of Studies of Instruments and Scale Development and Testing.^22^ This study was approved by the Research and Ethics Board at the University of Alberta (Edmonton, Alberta, Canada; Pro00139874).

## Results

In total, 9,448 patient records were retrieved. From the total records, 798 (8%) were excluded due to incomplete clinical data, leaving 8,650 records in the analytic sample. Patient characteristics for the analytic sample are reported in Table 1. Males made up 71% (n = 6,147) of the sample. The majority of patients (92%; n = 7,987) experienced blunt trauma. The next most common mechanism was penetrating trauma (6%; n = 551). Pediatric patients (ie, ≤18 years of age) represented nine percent (n = 733) of the sample, and geriatric patients (ie, >65 years of age) made up 28% (n = 2,418) of the sample.

**Table 1.** Patient Characteristics

Characteristic	Count (%)
**Sex**	
Male	6,147 (71%)
Female	2,503 (29%)
**Age**	
≤18 years	733 (9 %)
19–65 years	5,452 (63%)
>65 years	2,418 (28%)
Missing	47 (1%)
**Mode of Injury**	
Blunt	7,987 (92%)
Penetrating	551 (6%)
Burn	109 (1%)
Other	3 (0%)
**Discharge Status**	
Alive	7,849 (91%)
Dead	801 (9%)
**ICU Admission**	
Admitted	2,432 (28%)
Length of Stay, Median in Days (Q1, Q3)	6.0 (3, 12)
**Injury Severity Score (ISS)**	
Median (Q1, Q3)	18 (16, 25)
Severe ISS (≥16)	6,781 (78%)
**Trauma Center**	
Level 1	6,374 (74%)
Level 2	1,572 (18%)
Level 3	704 (8%)
**Gold Standard Classifications**	
Dead	193 (2%)
Expectant	166 (2%)
Immediate	2,528 (29%)
Delayed	5,763 (67%)
Total	8,650 (100%)

Abbreviations: ICU, intensive care unit; ISS, Injury Severity Score.

Using the reference standard criteria, a total of 2,528 patients (29%) were identified as having received an urgent life-saving intervention, meaning they met the definition of being a “Priority 1” patient. The most common intervention that “Priority 1” patients received was advanced airway protection (61%), followed by chest tube placement (38%); Table 2.

**Table 2.** Life-Saving Interventions among “Priority 1” Patients According to the Reference Standard Definition

Interventions	Count (%)
Escharotomy	0 (0%)
Chest Tube	969 (38%)
Advanced Airway Protection	1,545 (61%)
Blood Product Administration	417 (17%)
CPR	48 (2%)
Surgery	574 (23%)

Note: Some patients received more than one intervention, so percentages might not add to 100%.

Abbreviation: CPR, cardiopulmonary resuscitation.

**Figure 1.**
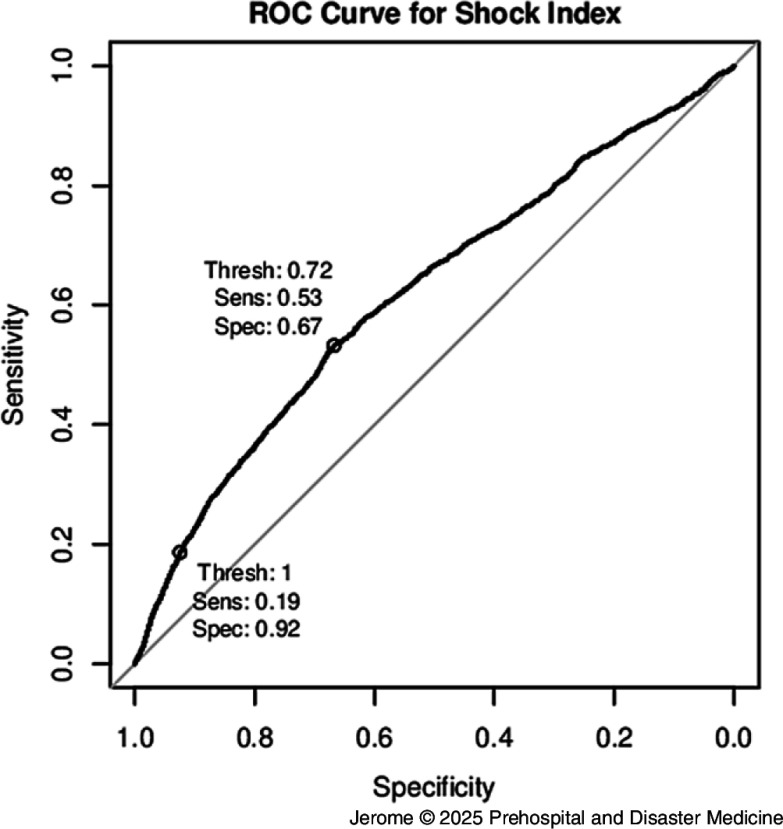
Receiver Operating Curve (ROC) for the Need of a Life-Saving Intervention.

Figure 1 presents the ROC curve for the SI. Area under the ROC curve (AUC) was 0.62 (95% bootstrap CI, 0.60-0.63). The SI threshold maximizing Youden’s index was 0.72 (95% bootstrap CI, 0.69-0.72). A total of 3,380 patients had an SI above 0.72. At this threshold, SI had a sensitivity of 0.53 (95% CI, 0.51-0.55) and a specificity of 0.67 (95% CI, 0.66-0.68; Table 3) for identifying “Priority 1” patients. A subgroup of 880 patients had an SI above 1.00 and, at this threshold, SI had a sensitivity of 0.19 (95% CI, 0.17-0.20) and a specificity of 0.92 (95% CI, 0.92-0.93).

**Table 3.** Performance of the Shock Index in Identifying “Priority 1” Patients

Tool	Sensitivity	Specificity	PPV	NPV
SI of 0.72	0.53 (0.51, 0.55)	0.67 (0.66, 0.68)	0.40 (0.38, 0.41)	0.78 (0.76, 0.79)
SI of 1	0.19 (0.17, 0.20)	0.92 (0.92, 0.93)	0.50 (0.47, 0.54)	0.73 (0.72, 0.74)

Note: Numbers in parentheses denote 95% confidence intervals. Shock Index = SBP/HR.

Abbreviations: SI, Shock Index; HR, heart rate; SBP, systolic blood pressure; PPV, positive predictive value; NPV, negative predictive value.

When stratified by injury type, the sensitivity of SI with a threshold of 0.72 was highest for penetrating trauma at 0.69 (95% CI, 0.64-0.73) and lowest for blunt trauma at 0.50 (95% CI, 0.48-0.52); Table 4. Similarly, the sensitivity of SI with a threshold of 1.00 was highest for penetrating trauma at 0.29 (95% CI, 0.25-0.34) and lowest for blunt trauma at 0.16 (95% CI, 0.15-0.18).

**Table 4.** Performance of the Shock Index in Identifying “Priority 1” Patients, Stratified by Injury Mechanism

Tool	Sensitivity	Specificity	PPV	NPV
Blunt				
SI threshold 1.00	0.16 (0.15, 0.18)	0.93 (0.92, 0.94)	0.45 (0.41, 0.49)	0.76 (0.75, 0.77)
SI threshold 0.72	0.50 (0.48, 0.52)	0.68 (0.67, 0.69)	0.35 (0.34, 0.37)	0.79 (0.78, 0.81)
Burn				
SI threshold 1.00	0.25 (0.15, 0.38)	0.78 (0.63, 0.88)	0.58 (0.37, 0.76)	0.46 (0.35, 0.57)
SI threshold 0.72	0.62 (0.48, 0.74)	0.37 (0.24, 0.52)	0.54 (0.42, 0.66)	0.44 (0.29, 0.60)
Penetrating				
SI threshold 1.00	0.29 (0.25, 0.34)	0.80 (0.73, 0.86)	0.78 (0.70, 0.84)	0.32 (0.28, 0.37)
SI threshold 0.72	0.69 (0.64, 0.73)	0.38 (0.31, 0.46)	0.73 (0.68, 0.77)	0.34 (0.27, 0.41)

Note: Numbers in parentheses denote 95% confidence intervals. Shock Index = SBP/HR.

Abbreviations: SI, Shock Index; HR, heart rate; SBP, systolic blood pressure; PPV, positive predictive value; NPV, negative predictive value.

When stratified by patient age, the sensitivity of SI with a threshold of 0.72 was highest for pediatric patients at 0.70 (95% CI, 0.63-0.75) and lowest for geriatric patients at 0.30 (95% CI, 0.25-0.34); Table 5. Similarly, the sensitivity of SI with a threshold of 1.00 was highest for pediatric patients at 0.29 (95% CI, 0.23-0.35) and lowest for geriatric patients at 0.08 (95% CI, 0.06-0.12).

**Table 5.** Performance of the Shock Index in Identifying Priority 1 Patients, Stratified by Patient Age

	Sensitivity	Specificity	PPV	NPV
Pediatric				
SI threshold 1.00	0.29 (0.23, 0.35)	0.76 (0.72, 0.80)	0.34 (0.28, 0.42)	0.71 (0.67, 0.75)
SI threshold 0.72	0.70 (0.63, 0.75)	0.35 (0.31, 0.39)	0.31 (0.27, 0.36)	0.73 (0.67, 0.78)
Adult				
SI threshold 1.00	0.20 (0.18, 0.21)	0.93 (0.92, 0.94)	0.60 (0.56, 0.64)	0.69 (0.68, 0.70)
SI threshold 0.72	0.57 (0.54, 0.59)	0.63 (0.61, 0.64)	0.44 (0.42, 0.46)	0.73 (0.72, 0.75)
Geriatric				
SI threshold 1.00	0.08 (0.06, 0.12)	0.97 (0.96, 0.98)	0.38 (0.29, 0.49)	0.83 (0.81, 0.85)
SI threshold 0.72	0.30 (0.25, 0.34)	0.83 (0.81, 0.85)	0.28 (0.24, 0.32)	0.85 (0.83, 0.86)

Note: Numbers in parentheses denote 95% confidence intervals. Shock Index = SBP/HR. Pediatric calculated as <18 years of age. Geriatric calculated as >65 years of age.

Abbreviations: SI, Shock Index; HR, heart rate; SBP, systolic blood pressure; PPV, positive predictive value; NPV, negative predictive value.

The PPV of SI was low with values of 0.40 (95% CI, 0.38-0.41) and 0.50 (95% CI, 0.47-0.54) for the thresholds of 0.72 and 1.00, respectively. Conversely, NPV was moderate at 0.78 (95% CI, 0.76-0.79) and 0.73 (95% CI, 0.72-0.74) for the same respective thresholds. The PPV was highest (at least 0.73 for either threshold) for penetrating injuries and NPV was highest (at least 0.83 for either threshold) for geriatric patients.

## Discussion

### Interpretation of SI Performance

This study assessed the performance of both an optimal SI threshold of 0.72 and a threshold of 1.00 in identifying “Priority 1” patients. The threshold of 1.00 was included because it is an attractive cutoff to use in clinical practice as it can be applied very easily by first responders. Unfortunately, the sensitivity of 0.19 for the threshold of 1.00 was much lower than the sensitivity of 0.53 obtained when using the optimal SI threshold of 0.72 determined by Youden’s index. While the SI threshold of 1.00 had low sensitivity, it demonstrated a high specificity (0.92). The low sensitivity means that the SI threshold of 1.00 cannot be safely used in isolation as a method of triage. The high specificity, however, means that it may be possible that SI values above 1.00 could be incorporated into future triage tools in order to improve their performance and limit the rate of under-triage.

### Comparison to Formal Triage Tools

Formal triage tools employ a variety of clinical variables (such as tachycardia, tachypnea, bradypnea, decreased Glasgow Coma Scale score, and lack of palpable peripheral pulses) to determine a triage classification for individual patients.^1–7^ Unfortunately, these relatively simple algorithms are still challenging to apply accurately, and providers often misclassify patients.^23–25^ Previous studies have also demonstrated that, even when they are applied correctly, these tools may fail to accurately identify traumatic patients who meet the reference standard definition of a “Priority 1” patient.^8–10^ If SI had a higher sensitivity than formal triage tools for identifying “Priority 1” patients, the SI could be considered as an alternate method of triage during MCI events. Both SI thresholds assessed in this study had sensitivities lower than the sensitivities of the triage tools MPTT (0.76) and BCD (0.70).^8^ The SI threshold of 0.72 out-performed the other four triage tools assessed in the reference study (START, Jump START, SALT, and RAMP).^8^ The SI threshold of 1.00 performed worse than all six triage tools assessed in the previous study. These results indicate that assessing SI does not out-perform existing tools for triaging trauma patients.

### Outcome Selection

The purpose of the triage process is to identify patients who may be critically unwell and may require timely life-saving interventions during MCI events. Triage assessments with a low sensitivity will not reliably identify “true positives” (ie, the patients who truly require these interventions). Failing to identify these critically unwell patients at initial assessment has a greater clinical impact than falsely identifying patients who do not actually need these life-saving interventions. Sensitivity was therefore chosen as the primary outcome in the evaluation of the performance of SI and the triage tools. Minimizing under-triage in an MCI is important for ensuring that the most severely injured patients receive timely, life-saving care, while balancing the risk of over-triage is essential to avoid overwhelming limited resources and compromising the overall efficiency of the response. Specificity, PPV, and NPV were measured as secondary outcomes to assess these elements of the performance of the triage tools.

### Previous Studies Assessing SI in Trauma

King, et al found that in trauma patients, an elevated SI increases the likelihood that a patient would have an ISS above 16, would require admission to the intensive care unit (ICU) lasting more than one day, would experience mortality within 24 hours, or would require a blood transfusion of more than two units.^15^ They calculated that the optimal SI thresholds for determining these outcomes were 1.10 for mortality within 24 hours, 0.71 for ISS above 16, 0.77 for ICU stay more than one day, and 0.85 for blood transfusion. The optimal SI value for any of the above measures was determined to be 0.83.^15^ Other than the threshold for mortality, these optimal thresholds are all similar to the optimal threshold of 0.72 calculated in this study. This suggests that despite its lack of sensitivity, the optimal threshold calculated in this study is in the appropriate range and likely represents the best SI threshold for identifying trauma patients who are critically unwell and require urgent life-saving interventions.

Both King, et al and Vernon, et al evaluated the ability of SI to identify trauma patients who died within 24 hours of their presentation to the ED.^12,15^ Prognosticating 24-hour mortality, however, is not the best outcome to employ when assessing the performance of triage tools. The purpose of triage is to determine which patients require immediate life-saving interventions. This is a different group of patients from those who are likely to die within the next day, despite receiving care. The outcome employed in this study – Lerner, et al’s definition of a “Priority 1 (Immediate)” patient – was developed to serve as a clinically relevant outcome to be employed when assessing the performance of triage tools. This study therefore provides a more valuable assessment of the performance of SI as a triage tool.

Scholl, et al showed that a SI threshold of 1.00 is more sensitive than massive transfusion scoring tools such as the Assessment of Blood Consumption (ABC score) at identifying trauma patients who will require massive transfusion protocols (MTPs).^13^ In their study, an SI threshold of 1.00 was assessed as having a sensitivity of 0.68 with a specificity of 0.81 for predicting MTP. In this study, both the optimal SI threshold of 0.72 and the threshold of 1.00 had significantly lower sensitivities than the sensitivity of SI reported by Scholl, et al. The reference standard criteria employed in this study included receiving a blood transfusion of ≥ four units of blood products within 24 hours as one of the life-saving interventions (Supplemental Materials; available only only). Scholl, et al defined MTP as transfusing >10 units of blood products within 24 hours. Their more restrictive criteria may account for the different findings between the two studies.

## Limitations

This study was a retrospective review of clinical data from a provincial trauma registry and is subject to the limitations common to retrospective investigations. The ATR dataset has the strength that it captures clinical data early in the patient’s care, including prehospital data when available. The registry only records a single set of prehospital vital signs, however, and the data input into the registry is subject to reporting error. Reporting error is more likely for data captured in the prehospital environment, where there are fewer personnel and where providing clinical care is sometimes prioritized over completing concurrent record keeping. It is also possible that clinical data recorded in the registry were measured after significant interventions were performed (ie, a patient was intubated early during their care, and the ATR reported post-intubation vital signs). Assuming that HR and blood pressure trend towards normalization after the performance of life-saving interventions, this could have artificially lowered the specificities calculated in this study.

A number of assumptions were made when working with the data retrieved in this study. One of the most significant assumptions is that all the procedures patients received were clinically indicated. The majority of the patients in the dataset experienced trauma outside of an MCI scenario where the mechanism of injury and the corresponding injury patterns may not be representative of those experienced by patients in an actual MCI. It is possible that some of the procedures that patients received could have been appropriately withheld or postponed, especially in an MCI situation where clinical resources are limited. Assumptions also had to be made to match the clinical data captured in the database to the definitions established in the triage tools and the reference standard criteria.

## Conclusion

The optimal SI level, with the highest sensitivity for identifying “Priority 1” patients, was determined to be 0.72. Even at this ideal threshold, however, SI did not out-perform existing MCI triage tools in identifying trauma patients who received urgent life-saving interventions.

## Supporting information

Jerome et al. supplementary material Jerome et al. supplementary material

## Supplementary Materials

To view supplementary material for this article, please visit https://doi.org/10.1017/S1049023X25101209
