# Supplementary material for: The Use of the Shock Index to Classify Patients During Mass-Casualty Incident Triage
Source: Prehosp Disaster Med. 2025 Jun 25;40(3):156–61. doi: 10.1017/S1049023X25101209 (PMC12237700; doi:10.1017/S1049023X25101209)
Supplement: Jerome et al. supplementary material [file S1049023X25101209sup001.docx]

**Supplementary Data**

Table 1 – Criteria Used to Determine Reference Standard Classifications Using Clinical Data Available in the ATR

| **Reference Standard Definitions [9]** | **Clinical Data Available in the ATR** |
| --- | --- |
| **DEAD** | |
| A lack of palpable pulse and/or respiratory effort (i.e. cardiac or respiratory arrest) at initial EMS assessment that is not responsive to airway positioning or needle decompression | - Record of prehospital cardiorespiratory resuscitation *or* - record of prehospital cardiac arrest |
| Lack of pulse or respiratory effort within 15 minutes of EMS arrival at scene | - Record of prehospital cardiorespiratory resuscitation *or* - record of prehospital cardiac arrest |
| **EXPECTANT** | |
| In patients aged 0 to 49 years old, third degree (full thickness) burns to >90% of the body | - Injury Type = Burn, *and* ISS = 75 |
| In patients over 50 years old, third degree (full thickness) burns to >80% of the body | - Injury Type = Burn, *and* ISS = 75 |
| Penetrating or blunt trauma to the head which crosses the midline with agonal respirations and/or no motor response decorticate posturing or decerebrate posturing (i.e. a motor GCS of 3 or less) | - Injury Type = Penetrating or Blunt, *and* record of CT HEAD, *and* earliest GCS Motor Score = 1-3, *and* Discharge Status = Dead |
| Uncontrolled haemorrhage that resulted in cardiac arrest prior to EMS transport | *Clinical data unavailable in the ATR* |
| **PRIORITY 1 (IMMEDIATE)** | |
| Neurologic, vascular, or haemorrhage-controlling surgery to the head, neck or torso performed within 4 hours of arrival to hospital | - If the patient’s disposition from the ED was directly to the operating room, *and* - There was a record of an operation performed on the same day that the patient presented to the ED and the operation was classified as either ““General Surgery”, “Trauma”, “Neurosurgery”, “Cardiothoracic”, “Critical Care”, Oromaxillo”, “”, “Vascular”, “Thoracic”, or “” |
| Limb-conserving surgery performed within 4 hours of arrival at hospital on a limb that was found to be pulseless distal to the injury prior to surgery | - If the patient’s disposition from the ED was directly to the operating room, *and* - There was a record of an operation performed on the same day that the patient presented to the ED and the operation was classified as either “Orthopedics” or “Plastic Surgery”” |
| Escharotomy performed on a patient with burns within 2 hours of arrival at a hospital | - If there was a record of the patient receiving an escharotomy at the referring hospital, or at the receiving trauma centre |
| Chest tube placed within 2 hours of arrival at hospital | - If there was a record of the patient receiving a chest tube in either the prehospital setting, in the receiving hospital, or in the ED of the receiving trauma centre |
| An advanced airway intervention (e.g. intubation, LMA, surgical airway) performed in the pre-hospital setting or within 4 hours of arrival at hospital | - If there was a record of the patient being intubated prehospital or at the referring facility, *or* - If any of the following procedures were performed in the prehospital, referring facility or in the ED of the receiving trauma centre: “King LT”, “LMA”, “Combitube”, “Oral Intubation”, “Raid Sequence Intubation”, or “Cricothyroidotomy” |
| IV vasopressors administered within 2 hours of arrival at hospital | - The use of IV vasopressors is not recorded in the ATR |
| Arrived in the ED with uncontrolled haemorrhage | - If the patient received 4 units or more of blood products in the referring facility, and/or within the first 24h of presentation to the trauma centre, *or* - If the patient had “surgery for hemorrhage control” |
| Patient who required EMS initiation of CPR (i.e. had a cardiac arrest) during transport, in the ED, or within 4 hours of arrival at a hospital | - If there was a record of CPR being performed at either the referring hospital, or in the ED of the receiving trauma hospital |
| **PRIORITY 3 (MINIMAL)** | |
| Discharged from the ED with no X-rays or an extremity X-ray that was negative or showed an uncomplicated fracture (i.e. a closed extremity fracture without significant displacement or neurovascular compromise); no laboratory testing; received only simple wound repair (single layer suturing only); and received no medications intravenously (does not include fluids), or inhaled (does not include oxygen) from EMS or in the hospital | *Due to the ATR’s inclusion criteria for the ATR, it was deemed that all patients in the database would have injuries too significant to meet this definition* |
| **PRIORITY 2 (DELAYED)** | |
| All remaining patients | - All remaining patients |
